# TAK1 Regulates Cartilage and Joint Development via the MAPK and BMP Signaling Pathways

**DOI:** 10.1002/jbmr.79

**Published:** 2010-03-08

**Authors:** Lea M Gunnell, Jennifer H Jonason, Alayna E Loiselle, Anat Kohn, Edward M Schwarz, Matthew J Hilton, Regis J O'Keefe

**Affiliations:** Department of Orthopaedics and Rehabilitation, Center for Musculoskeletal Research, University of RochesterRochester, NY, USA

**Keywords:** TAK1, MAPK, BMP, cartilage, joint

## Abstract

The importance of canonical transforming growth factor β (TGF-β) and bone morphogenetic protein (BMP) signaling during cartilage and joint development is well established, but the necessity for noncanonical (SMAD-independent) signaling during these processes is largely unknown. TGF-β activated kinase 1 (TAK1) is a MAP3K activated by TGF-β, BMP, and other mitogen-activated protein kinase (MAPK) signaling components. We set out to define the potential role for noncanonical, TAK1-mediated signaling in cartilage and joint development via deletion of *Tak1* in chondrocytes (*Col2Cre;Tak1*^*f/f*^) and the developing limb mesenchyme (*Prx1Cre;Tak1*^*f/f*^). Deletion of *Tak1* in chondrocytes resulted in novel embryonic developmental cartilage defects including decreased chondrocyte proliferation, reduced proliferating chondrocyte survival, delayed onset of hypertrophy, reduced *Mmp13* expression, and a failure to maintain interzone cells of the elbow joint, which were not observed previously in another *Col2Cre;Tak1*^*f/f*^ model. Deletion of *Tak1* in limb mesenchyme resulted in widespread joint fusions likely owing to the differentiation of interzone cells to the chondrocyte lineage. The *Prx1Cre;Tak1*^*f/f*^ model also allowed us to identify novel columnar chondrocyte organization and terminal maturation defects owing to the interplay between chondrocytes and the surrounding mesenchyme. Furthermore, both our in vivo models and in vitro cell culture studies demonstrate that loss of *Tak1* results in impaired activation of the downstream MAPK target p38, as well as diminished activation of the BMP/SMAD signaling pathway. Taken together, these data demonstrate that TAK1 is a critical regulator of both MAPK and BMP signaling and is necessary for proper cartilage and joint development. © 2010 American Society for Bone and Mineral Research.

## Introduction

Limb development is a complicated process culminating in the formation of a highly functional limb skeleton composed of cartilage, bone, connective tissues, and mobile joints. The limb skeleton originally derives from mesenchymal cells of the lateral-plate mesoderm that migrate, condense, proliferate, differentiate, and undergo terminal maturation. As condensed mesenchymal cells undergo the process of chondrogenesis, several layers of cells at prospective joint regions adopt a nonchondrogenic fate, forming the mesenchymal interzone. Interzone cells ultimately give rise to the synovium, joint capsule, and articular cartilage tissues of adult synovial joints.(1) Genetic studies have implicated several signaling factors in the proper formation and maintenance of joints, including *Wnt9a, Wnt4, Erg, Noggin, Gdf5, Bmpr1a*, and *Bmpr1b*.(1–8)

Mesenchymal cells outside interzone regions complete the process of chondrogenesis primarily through the activities of Sox family transcription factors—*Sox9, Sox5*, and *Sox6*.(9,10) After initial formation of the individual cartilage rudiments, coupled processes of chondrocyte proliferation and maturation are responsible for longitudinal growth. Chondrocyte proliferation serves as the initial driving force of longitudinal growth, but ultimately proliferating cells near the center of cartilage elements withdraw from the cell cycle and undergo chondrocyte maturation or hypertrophic differentiation. Progression of chondrocyte maturation can be followed via examination of key markers of maturation: Prehypertrophic and early hypertrophic chondrocytes express *Indian hedgehog* (*Ihh*) and *Runx2*, whereas hypertophic chondrocytes generating a mineralizing matrix are characterized by *type X collagen* (*Col10a1*) expression, and finally, the most terminally hypertrophic chondrocytes express *matrix metalloproteinase 13* (*Mmp13*), an enzyme that controls degradation and removal of the cartilage matrix.(11) Completion of chondrocyte maturation is necessary for proper vascular invasion, establishment of the marrow cavity, and normal growth plate development.

The transforming growth factor β (TGF-β) superfamily represents a group of molecules critical for normal skeletal growth and development. Recent mouse genetic studies have provided important insights into the contributions of each of these molecules to the processes of chondrogenesis, chondrocyte maturation, and joint morphogenesis. Mouse models lacking the TGF-β type II receptor in chondrocytes (*Col2Cre;Tgfbr2*^*f/f*^) exhibit defects in the postnatal regulation of chondrocyte maturation primarily within the axial skeleton.(12) Deletion of *Tgfbr2* in early limb mesenchyme (*Prx1Cre;Tgfbr2*^*f/f*^) resulted in delayed cartilage formation, reduced chondrocyte proliferation, and malformation of joints within the digits.(13) Bone morphogenetic protein (BMP) signaling molecules are also known to be important regulators of chondrogenesis, chondrocyte differentiation, joint formation, proliferation, and apoptosis.(4,14–20). Mice lacking BMP receptors 1a and 1b within cartilage (*Col2Cre;Bmpr1a*^*f/f*^, *Bmpr1b*^*+/−*^, and *Col2Cre;Bmpr1a* ^*f/f*^*;Bmpr1b*^*−/−*^) or lacking canonical BMP targets Smads 1 and 5 within cartilage (*Col2Cre;Smad1*^*f/f*^*;Smad5*^*f/f*^) have reduced chondrocyte proliferation, delayed onset of chondrocyte maturation, reduced proliferating chondrocyte survival, and delayed progression of terminal maturation.(16,17) Conditional deletion of the BMP ligands, BMP-2 and BMP-4, using the *Prx1Cre* transgene (*Prx1Cre;Bmp2*^*f/f*^*;Bmp4*^*f/f*^) resulted in shorter and thinner limbs, delayed differentiation and marrow cavity formation, disorganized clearance of hypertrophic chondrocytes, joint fusion of the stylopod and zeugopod elements with little effect on the autopod, and incomplete bone formation.(14) Conventional deletion of another BMP family member, *Gdf5,* also demonstrated effects on the limb skeleton, resulting in decreased limb length and various joint fusions/abnormalities.(4,8,15)

TGF-β superfamily signaling is initiated when ligand binds to the type II serine/threonine kinase receptor, leading to activation of the type I receptor and induction of downstream signaling mechanisms.(21) Canonical TGF-β and BMP signaling occurs via activation of receptor Smads (R-Smads) 2/3 and 1/5/8, respectively. While the TGF-β and BMP canonical pathways play critical and differential roles during chondrogenesis and chondrocyte maturation, in vitro studies have demonstrated recently a role for noncanonical signaling via the activation of MAPKs. Noncanonical signaling by the TGF-β and BMP pathways is thought to activate MAPK signaling via induction of transforming growth factor β activated kinase 1 (TAK1).(21) On activation, TAK1 directly phosphorylates MKK 3/6, leading to the subsequent phosphorylation and activation of p38.(22,23)

The importance of TAK1 activation by TGF-β and BMP in cartilage remains unclear. In vitro data suggest that noncanonical signaling via MAPKs can regulate chondrogenesis, hypertrophic differentiation, and proliferation via TGF-β-, Gdf5-, and BMP-mediated activation of p38.(24–29) Recent studies aimed directly at MAPK pathway components have suggested that both overexpression and inhibition of the MAPK factors can generate similar in vitro chondrocyte phenotypes, leading investigators to question whether MAPK signaling supports a single event or if it is the balance of its activity that is important.(24,27) Use of these various gain- and loss-of-function mouse models has similarly demonstrated that activation or inhibition of the MAPK pathway results in analogous cartilage and bone phenotypes that manifest primarily during postnatal development. Loss-of-function mouse models, dominant-negative (dn) P38 and Atf2- deficient mice, have demonstrated that disruption of MAPK signaling results in postnatal chondrodysplasia, decreased chondrocyte proliferation, and decreased chondrocyte survival.(30–32) Meanwhile, transgenic mice overexpressing constitutively active MKK6 in chondrocytes are dwarfed, have decreased chondrocyte proliferation, delayed onset of hypertrophic differentiation during embryonic development, and a shortened zone of hypertrophic chondrocytes.(33) Recently, Shim and colleagues(34) showed the importance of TAK1 in postnatal chondrocytes when they analyzed a group of *Col2Cre;Tak1*^*f/f*^ mice. Their analyses indicated that loss of *Tak1* in chondrocytes resulted in postnatal skeletal defects, including decreased chondrocyte proliferation, increased apoptosis, elbow dislocations, and deficient BMP and MAPK signaling. Despite the clear postnatal skeletal phenotypes presented by the disruption of MAPK signaling components, including TAK1, the roles of these pathways have not been thoroughly examined during embryonic skeletal development. Therefore, we set out to determine whether TAK1 represents an important upstream component of the MAPK and TGF-β/BMP signaling pathways during embryonic cartilage and joint development by deleting floxed *Tak1* alleles in cartilage using a different *Col2Cre* transgenic line and in early limb mesenchyme using the *Prx1Cre* transgene.

## Materials and Methods

*Tak1*^*f/f*^ mice were generated as described previously.(35) *Tak1*^*f/f*^ mice were crossed with *Col2Cre* transgenic mice(36) to delete *Tak1* floxed alleles within the developing cartilage. The *Col2Cre;Tak1*^*f/f*^ mice are referred to as *Tak1*^*Col2*^ *mutants*. *Tak1* deletion within the early limb mesenchyme was performed by crossing *Tak1*^*f/f*^ mice with the *Prx1Cre* transgenic mouse.(37) *Prx1Cre;Tak1*^*f/f*^ mice are referred to as *Tak1*^*Prx*^ *mutants*.

### Skeletal preparation, histology, in situ hybridization, and immunohistochemistry

Whole embryos for alcian blue/alizarin red skeletal staining were prepared as described previously.(38) Embryos harvested for histology were dissected in PBS, fixed in 10% neutral buffered formalin (NBF), decalcified in 14% EDTA, processed in paraffin, and embedded prior to sectioning at 6 µm. Hematoxylin and eosin (H&E), safranin O, and fast green or alcian blue, hematoxylin, and orange G stains were performed on various sections to identify tissue/cell morphology of the limbs. In situ hybridization was performed on longitudinal forelimb sections using ^35^S radiolabeled probes as described previously.(39) Many of our antisense probes have been described previously.(3,38–40) Riboprobes not previously described were generated from the following cDNA clones: *Wnt9a* (30435371, Open Biosystems, Huntsville, AL, USA), *Erg* (40131153, Open Biosystems), *Gdf5*,(15) and *Agc1* (5345931, Open Biosystems). Immunohistochemistry was performed for p-Smad 1/5/8 (1:100 dilution; Cell Signaling, Danvers, MA, USA) and type II collagen (Thermo Scientific, Freemont, CA, USA) on paraffin sections using standard protocols.

### Chondrocyte proliferation and apoptosis

For cell proliferation assays, pregnant female mice were administered BrdU via i.p. injection 2 hours prior to killing. Embryos were dissected, fixed, decalcified, and processed as described earlier. BrdU immunohistochemistry was performed on three limb sections for three separate embryos of each genotype using the Invitrogen BrdU Kit (Invitrogen, Carlsbad, CA, USA). Proliferation rate is reported as the percentage of BrdU-positive nuclei in the proximal humerus relative to wild-type littermates. Changes in chondrocyte apoptosis were determined using the In Situ Cell Death Kit (Roche, Indianapolis, IN, USA) according to manufacturer's protocol and reported as absolute number of terminal deoxynucleotidyl transferase–mediated deoxyuridine triphosphate–biotin nick end labeling (TUNEL)^+^ apoptotic cells in the proximal humerus. Cells were counted and averaged for three separate wild-type and mutant sections from three independent litters.

### Western blot analysis of limb cartilage

Dissected cartilage for Western blotting was lysed using RIPA buffer (150 mM NaCl, 1.0% Triton-X, 0.5% sodium deoxycholate, 0.1% SDS, 50 mM Tris. pH 8.0) containing minicomplete protease inhibitor (Roche) and PhosSTOP phosphatase inhibitor tablets (Roche). Whole-cell lysate was quantified, normalized, and run on 10% Bis-Tris gels (Invitrogen). Blots were incubated with primary antibodies including total TAK1 (Cell Signaling) and β-actin (Sigma). Blots then were washed in Tris-buffered saline with Tween-20 (TBST), incubated in secondary antibody, and visualized using chemiluminescence (Pierce, Rockford, IL, USA).

### Cell culture and signaling

Primary sternal chondrocytes were isolated from 2- to 3-day-old *Tak1*^*f/f*^ mice as described previously(41–43) or from E14.5 cartilage elements of the limb. All cells were seeded as follows: 1 × 10^6^ cells per well in a six-well plate and maintained in DMEM containing 10% FBS and 1% penicillin/streptomycin. In vitro deletion was performed using Ad5-CMV-GFP (control) or Ad5-CMV-Cre viruses (Baylor Vector Development, Houston, TX, USA). Cells were infected for 48 hours and allowed to recover overnight before treatment. For alkaline phosphatase staining, cells were fixed in 4% paraformaldehyde, washed, and stained using NTB/BCIP (Thermo Scientific).

Rat chondrosarcoma (RCS) cells were cultured in DMEM containing 10% FBS and 1% penicillin/streptomycin.(44) For Western blotting, cells were serum-starved overnight, pretreated with the *cis*-enol resorcylic acid lactone–selective TAK1 inhibitor (TI-2), LLZ1640-2 (Bioaustralis, Smithfield, NSW, Australia),(45) the inactive homologue (TI-4), LLZ1640-4, or DMSO (Sigma) and then treated for 60 minutes with 100 ng/mL of BMP2 (Peprotech, Rocky Hill, NJ, USA). Cells were lysed using NP-40 lysis buffer (150 mM NaCl, 1% NP-40, 50 mM Tris-HCl, pH 8.0) containing mini-complete protease inhibitor (Roche), PhosSTOP phosphatase inhibitor tablets (Roche), 1 mM sodium orthovanadate, 10 mM NaF, and 0.5 mM okadaic acid. Lysates were run on 10% Bis-Tris gels (Invitrogen), and antibodies used included TAK1, pSmad 1/5/8, pP38, tP38, atubulin (Cell Signaling), and total Smad 1/5/8 (Santa Cruz Biotechnology, Santa Cruz, CA, USA). Gene expression studies used RNA extracted with Trizol reagent (Invitrogen) according to manufacturer's protocol. Real-time RT-PCR was performed on a RotorGene RT Machine (Qiagen, Valencia, CA, USA) using 1 µL of cDNA and PerfeCTa SYBR Green reagent (Quanta BioSciences, Gaithersburg, MD, USA). For reporter assays, cells were transfected with 12×-SBE luciferase reporter(46) using Fugene-HD (Roche) according to the manufacturer's protocol. Cells were serum-starved overnight and pretreated with TAK1 inhibitor for 30 minutes prior to treatment with 100 ng/mL of BMP2. Luciferase activity was measured 24 hours later using the Dual-Promoter Luciferase Assay Kit (Promega) and normalized to *Renilla* luciferase. Triplicates of each treatment group were analyzed, and statistical significance was determined by *t* test.

## Results

### Loss of *Tak1* within cartilage results in neonatal lethality

*Tak1* is expressed throughout the cartilage and surrounding tissues during endochondral bone development and during in vitro chondrocyte maturation (Supplemental Fig. 1*A, B*). Interestingly, cartilage deletion of *Tak1* floxed alleles using a *Col2Cre* transgenic line resulted in neonatal lethality immediately following birth. X-ray and micro–computed tomographic (µCT) analyses of newborn pups on postnatal day 0 (*P*_0_) demonstrated that *Col2Cre;Tak1*^*f/f*^ (*Tak1*^*Col2*^) mutant mice exhibit decreased skeletal growth and have a significantly smaller ribcage compared with littermate controls (Supplemental Fig. 2*A*). Alcian blue and alizarin red skeletal staining for cartilaginous components of the airway revealed an undermineralized hyoid and a fusion of the hyoid to the larynx in *Tak1*^*Col2*^ mutant pups (Supplemental Fig. 2*B*). These data and the visual observation of pups at birth indicated that *Tak1*^*Col2*^ mutants do not survive owing to respiratory distress.

### *Tak1*^*Col2*^ mutants display defects in cartilage proliferation, maturation, and apoptosis

**Fig. 1:**
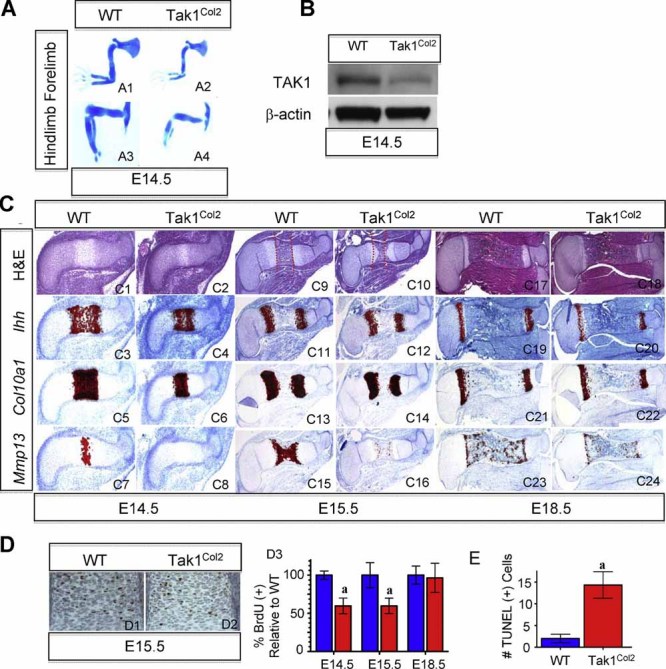
TAK1 regulates chondrocyte proliferation, maturation, and immature chondrocyte survival. (*A*) Alcian blue skeletal staining of forelimbs (*A*1, *A*2) and hindlimbs (*A*3, *A*4) from wild-type and *Tak1^Col2^* embryos at E14.5. (*B*) Lysates from cartilage of E14.5 humerus immunoblotted using anti-TAK1 antibody. (*C*) Hematoxylin and eosin staining of E14.5 (*C*1, *C*2), E15.5 (*C*9, *C*10), and E18.5 (*C*17, *C*18) humerus sections. (*C*9, *C*10) Red dashed lines indicate the chondroosseous junction. In situ hybridization for *Ihh, Col10a1*, and *Mmp13* at E14.5 (*C*3–*C*8), E15.5 (*C*11–*C*16), and E18.5 (*C*19–*C*24). (*D*) Representative IHC for BrdU of E15.5 wild-type (*D*1) and *Tak1^Col2^* (*D*2) embryos. (*D*3) Quantification of BrdU of cells in the proximal humerus of wild-type (*blue*) and *Tak1^Col2^* (*red*) embryos at E14.5, E15.5, and E18.5. (*D*2) Quantification of TUNEL^+^ cells in the proximal humerus of wild-type and *Tak1^Col2^* embryos at E18.5 (^a^*p* < .05 versus wild type).

Examination of limbs isolated from alcian blue– and alizarin red–stained embryos at 14.5 days of gestation (E14.5) clearly demonstrated a decrease in the size of both forelimb (Fig. 1*A*, *A*1 and *A*2) and hindlimb (Fig. 1*A*, *A*3 and *A*4) elements of *Tak1*^*Col2*^ mutants compared with wild-type littermate controls. Western blot analysis performed on protein extracted from E14.5 humeri cartilages verified deletion of *Tak1* in chondrocytes of the developing skeleton, although low levels of TAK1 protein remained detectable likely owing to contaminating perichondrium and surrounding soft tissues inadequately removed during isolation procedures (Fig. 1*B*). H&E staining of humerus sections at E14.5 revealed a shorter limb in *Tak1*^*Col2*^ mutants and a much smaller hypertrophic region (Fig. 1*C*, *C*1 and *C*2). Molecular analyses of chondrocyte maturation via in situ hybridization demonstrated a delay in the onset of hypertrophy, as evidenced by the decreased domain of expression for the prehypertrophic marker *Ihh* (Fig. 1*C*, *C*3 and *C*4), the hypertrophic marker *Col10a1* (Fig. 1*C*, *C*5 and *C*6), and the complete absence of the terminal hypertrophic marker *Mmp13* (Fig. 1*C*, *C*7 and *C*8). H&E staining of E15.5 humerus sections demonstrated that the primary center of ossification had begun to form in both wild-type and *Tak1*^*Col2*^ mutant limbs (Fig. 1*C*, *C*9 and *C*10), but uniform development of the marrow cavity in the *Tak1*^*Col2*^ mutant was incomplete owing to a continuous delay in chondrocyte maturation (Fig. 1*C*, *C*9 abd *C*10, *red dashed contours*). Molecular analysis of the maturational markers *Ihh, Col10a1*, and *Mmp13* demonstrated that the spatial expression domains of *Ihh* (Fig. 1*C*, *C*11 and *C*12) and *Col10a1* (Fig. 1*C*, *C*13 and *C*14) are less well separated in *Tak1*^*Col2*^ mutants compared with littermate controls, whereas *Mmp13* expression (Fig. 1*C*, *C*15 and *C*16) is markedly reduced in *Tak1*^*Col2*^ mutant limbs at E15.5. To determine if the delayed marrow development was due to impaired vascularization, we performed in situ hybridization to assess *angiopoietin 1* (*Angpt1*) expression within the developing marrow cavity of wild-type and *Tak1*^*Col2*^ mutant limbs. However, the results were not remarkable (data not shown), suggesting that vascularization of the primary center of ossification occurred normally, and the primary defect is in chondrocyte maturation. Interestingly, analyses of E18.5 histologic sections and in situ hybridization for the chondrocyte maturation markers demonstrated a relatively normal appearance of *Ihh* and *Col10a1* expression domains in *Tak1*^*Col2*^ mutants at E18.5 (Fig. 1*C*, *C*17 through *C*22) as well as the distinct presence of *Mmp13* expression at the chondroosseous junction, although *Mmp13* expression remained slightly reduced (Fig. 1*C*, *C*23 amd *C2*4), and the overall growth of the elements still was delayed.

To further address cartilage growth discrepancies between *Tak1*^*Col2*^ mutants and littermate controls, we performed BrdU and TUNEL labeling experiments on humeral sections of E14.5, E15.5, and E18.5 embryos. A representative section of BrdU immunohistochemistry (IHC) in the proliferative cells of the proximal humerus demonstrated that loss of *Tak1* leads to decreased chondrocyte proliferation at E15.5 (Fig. 1*D*, *D*1 and *D*2). Quantification of BrdU incorporation at E14.5 and E15.5 revealed that the loss of *Tak1* results in a significant decrease (40%) in chondrocyte proliferation, but by E18.5, chondrocyte proliferation is restored to levels near that of wild-type littermate controls (Fig. 1*D*, *D*3). TUNEL labeling studies further demonstrated that *Tak1*^*Col2*^ mutants exhibit significant elevations in resting/proliferating zone chondrocyte apoptosis of the developing humerus at E14.5 and E18.5 (Fig. 1*E*; and data not shown). Collectively, these data demonstrate that loss of *Tak1* in cartilage resulted in early delays in chondrocyte maturation, reductions in chondrocyte proliferation, and increased levels of immature chondrocyte apoptosis.

### *Tak1*^*Prx*^ mice exhibit delays in both the onset and progression of chondrocyte maturation

Owing to the neonatal lethality and interesting cartilage phenotypes of the *Tak1*^*Col2*^ mice, we generated *Prx1Cre;Tak1*^*f/f*^ mice (*Tak1*^*Prx*^) to determine whether TAK1 controls endochondral ossification in a broader context during both embryonic and postnatal development. While the *Prx1Cre* transgene targets both the cartilage and surrounding tissues derived from the early limb mesenchyme,(37) it remains restricted primarily to the appendicular skeleton and thus would avoid any potential respiratory complications responsible for the neonatal death of *Tak1*^*Col2*^ mice. Many of these animals survive to the weaning stage (3 to 4 weeks), although additional skeletal anomalies affect their long-term feeding and survival (Supplemental Fig. 2*C*).

**Fig. 2:**
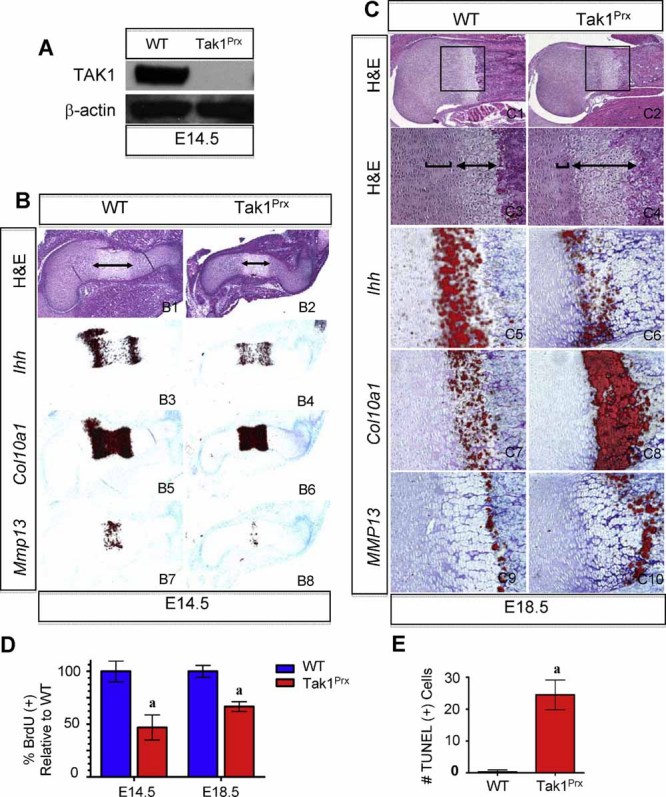
TAK1 indirectly regulates columnar chondrocyte organization and the progression of terminal maturation. (*A*) Immunoblot for TAK1 protein isolated from humerus cartilage of E14.5 wild-type and *Tak1^Prx^* embryos. (*B*) Histology and in situ hybridization of E14.5 wild-type and *Tak1^Prx^* humerus sections for H&E (*B*1, *B*2), *Ihh* (*B*3, *B*4), *Col10a1* (*B*5, *B*6), and *Mmp13* (*B*7, *B*8). (*C*1–*C*4) Double-sided arrows indicate hypertrophic region. H&E staining of proximal humerus from wild-type (*C*1, *C*3) and *Tak1^Prx^* (*C*2, *C*4) embryos at E18.5. Black boxes (*C*1, *C*2) indicate region of ×20 magnification. (*C*3, *C*4) Brackets denote height of columnar zone of chondrocytes. In situ hybridization of E18.5 wild-type and *Tak1^Prx^* humerus sections for *Ihh* (*C*5, *C*6), *Col10a1* (*C*7, *C*8), and *Mmp13* (*C*9, *C*10). (*D*) BrdU incorporation of E14.5 and E18.5 wild-type (*blue bars*) and *Tak1^Prx^* (*red bars*) embryos. (*E*) Quantitation of TUNEL^+^ cells in the proximal humerus at E18.5 (^a^*p* < .05 versus wild type).

We first analyzed the limb skeleton phenotypes of *Tak1*^*Prx*^ mutant mice at E14.5 and E18.5. *Prx1Cre* mediated deletion of *Tak1* was verified by immunoblotting for total TAK1 protein using lysates obtained from isolated limb cartilage elements of E14.5 embryos (Fig. 2*A*). H&E staining (Fig. 2*B*, *B*1 and *B*2), as well as in situ hybridization, on humeral sections from E14.5 wild-type and *Tak1*^*Prx*^ mutants supports a role for TAK1 in promoting the onset of chondrocyte hypertrophy because the expression domains of *Ihh* (Fig. 2*B*, *B*3 and *B*4), *Col10a1* (Fig. 2*B*, *B*5 and *B*6), and *Mmp13* (Fig. 2*B*, *B*7 and *B*8) were again both markedly reduced in size and level of expression. Interestingly, histologic examination of *Tak1*^*Prx*^ embryos at E18.5 indicated that in addition to the early delays in the onset of chondrocyte hypertrophy observed at E14.5 (Fig. 2*B*), loss of *Tak1* in surrounding mesenchymal tissues delayed the progression of chondrocyte maturation toward the terminal hypertrophic state, which was not observed in the *Tak1*^*Col2*^ embryos at this stage. H&E staining clearly demonstrated that E18.5 *Tak1*^*Prx*^ mutants have thinner cartilage elements and surrounding perichondrium, a disorganized and shortened columnar zone of chondrocytes (*brackets*), and an increased size of the hypertrophic zone (*double-headed arrows*) (Fig. 2*C*, *C*1 through *C*4). In situ hybridization for chondrocyte maturation markers was performed on the E18.5 humeral sections of *Tak1*^*Prx*^ and wild-type embryos. *Tak1*^*Prx*^ mutants exhibited reduced *Ihh* expression (Fig. 2*C*, *C*5 and *C*6), expansion of the *Col10a1* expression domain (Fig. 2*C*, *C*7 and *C*8) corresponding to the increased size of the hypertrophic region, and an expanded domain of *Mmp13* expression within the terminally hypertrophic chondrocyes at the chondroosseus junction (Fig. 2*C*, *C*9 and *C*10). Assessment of BrdU incorporation at E14.5 and E18.5 in *Tak1*^*Prx*^ embryos reveals that in addition to the decreased proliferation seen at E14.5 in *Tak1*^*Col2*^ embryos, loss of *Tak1* in limb mesenchyme results in a decrease in proliferation that persists at E18.5 (Fig. 2*D*). Similar to *Tak1*^*Col2*^ mutants, *Tak1*^*Prx*^ mice exhibited increased apoptosis in proliferating chondrocytes without any apparent change in apoptosis at the ends of the growth plate (Fig. 2*E*). Taken together, these data argue that TAK1 not only regulates chondrocyte proliferation, immature chondrocyte survival, and the onset of chondrocyte hypertrophy in cartilage but also controls the organization of columnar chondrocytes and the progression of chondrocyte terminal maturation via signaling within adjacent mesenchymal derived tissues.

### Loss or inhibition of TAK1 delays normal and BMP-induced chondrocyte maturation

**Fig. 3:**
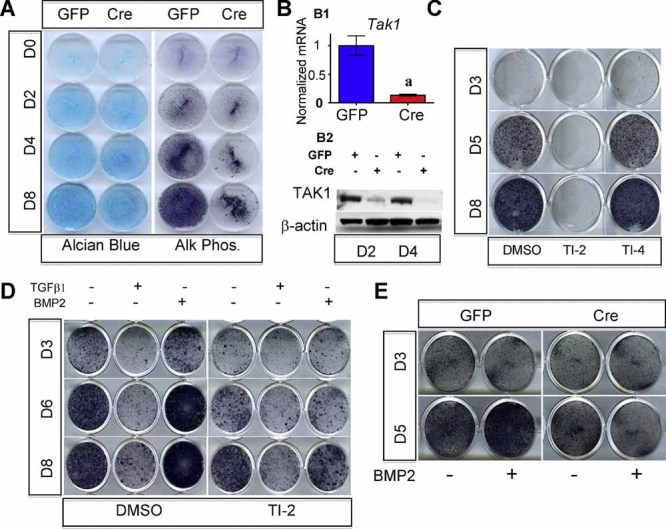
TAK1 regulates normal and BMP-mediated chondrocyte maturation. (*A*) Alcian blue and alkaline phosphatase (AP) staining of sternal chondrocytes isolated from *Tak1^f/^*^f^ pups and infected with Ad5-CMV-GFP (control) or Ad5-CMV-Cre. (*B*) RT-PCR for exon 1 of the *Tak1* gene on mRNA isolated from primary sternal chondrocytes (*B*1). Immunoblot for total TAK1 in lysates isolated from primary sternal chondrocytes after infection (*B*2). (*C*) Wild-type primary sternal chondrocytes treated daily with DMSO, 3 µM TI-2 (LLZ1640-2), or 3 µM TI-4 (LLZ1640-4) and stained for AP on indicated days. (*D*) AP staining of wild-type primary sternal chondrocytes treated every other day with DMSO or TI-2 (3 µM) and either no treatment, TGF-β (5 ng/mL), or BMP-2 (100 ng/mL). (*E*) AP staining of Ad-GFP- and Ad-Cre-infected primary sternal chondrocytes in untreated and BMP-2-treated (100 ng/mL) culture.

To further define the role of TAK1 in regulating chondrocyte development, we performed in vitro chondrocyte maturation experiments using *Tak1*^*f/f*^ sternal chondrocytes infected with *Cre* and control *Gfp* adenoviruses (Ad5-CMV-Cre-GFP and Ad5-CMV-GFP) or wild-type sternal chondrocytes treated with a pharmacologic TAK1 inhibitor or inactive homologue. The sternal chondrocyte model is a well-developed in vitro model of chondrocyte maturation(41–43) that was well suited for our study because cartilage in the sternum of *Tak1*^*Col2*^ mutants demonstrates maturational defects similar to those noted in *Tak1*^*Col2*^ developing limbs (Supplemental Fig. 3*B*). Alkaline phosphatase staining revealed a significant delay in chondrocyte maturation, as evidenced by the decreased staining over an 8-day differentiation period in *Tak1-*deleted cultures relative to Green Fluorescent Protein (GFP) controls (Fig. 3*A*). In order to verify that this difference in staining and maturation was not due to an absence of cells or a change in cell phenotype, we also stained the cultures with alcian blue, demonstrating that *Tak1-*deleted cultures maintained cell viability and the ability to produce matrix (Fig. 3*A*). The efficacy of the *Cre-*mediated deletion was approximately 85%, as determined by RT-PCR (Fig. 3*B*, *B*1) using primers designed to exon 1 of *Tak1.* Additionally, immunoblots for total TAK1 on whole-cell lysates following *Cre* infection demonstrated a near-complete loss of TAK1 protein (Fig. 3*B*, *B*2). Similar chondrocyte maturation experiments also were performed using daily treatments of the selective TAK1 inhibitor LLZ1640-2 (TI-2), the inactive homologue LLZ1640-4 (TI-4), or DMSO as an additional negative control. Treatment of sternal chondrocyte cultures with the TI-2 inhibitor (3 µM) displayed a robust suppression in chondrocyte maturation compared with either DMSO or TI-4 inactive homologue (3 µM) treatments (Fig. 3*C*). Identical effects were observed using primary chondrocytes isolated from E14.5 cartilage elements and treated with the TI-2 TAK1 inhibitor (Supplemental Fig. 4*A*). Treatment of sternal chondrocyte cultures with 3 µM TI-2 every other day (Fig. 3*D*) or with daily TI-2 treatments at reduced concentrations (data not shown) resulted in a less severe suppression of chondrocyte maturation.

To determine if TAK1 is required for TGF-β superfamily signaling and the subsequent regulation of chondrocyte maturation, we treated sternal chondrocyte cultures with either TI-2 or DMSO in the presence or absence of TGF-β1 (5 ng/mL) or BMP-2 (100 ng/mL) recombinant proteins. In order to visualize the BMP and TGF-β effects on alkaline phosphatase (AP) staining, we treated cultures with 3 µM TI-2 every other day. AP staining demonstrated that DMSO control cultures underwent normal chondrocyte maturation during the 8-day differentiation period. Treatment with TGF-β1 significantly impaired AP staining and chondrocyte maturation, whereas BMP-2 treatment enhanced or accelerated chondrocyte maturation compared with the DMSO-treated control (Fig. 3*D*). Treatment of these cultures with TI-2 resulted in decreased AP staining in both the presence and absence of BMP-2 (Fig. 3*D*). Interestingly, when the same TAK1 inhibitor treatments were performed in the presence of TGF-β1, TI-2 did not affect the ability of TGF-β1 to suppress chondrocyte maturation (Fig. 3*D*). Similar experiments also were performed using *Tak1*^*f/f*^ sternal chondrocytes infected with *Cre* and control *Gfp* adenoviruses. *Cre-*mediated deletion of *Tak1* resulted in impaired normal and BMP-2-induced chondrocyte maturation, as measured by AP staining, during a 5-day maturation period (Fig. 3*E*). All these data indicate that loss of *Tak1* or inhibition of TAK1 function disrupts BMP- but not TGF-β-mediated effects on chondrocyte maturation.

### Loss or inhibition of TAK1 impairs both MAPK and BMP signaling pathways

**Fig. 4:**
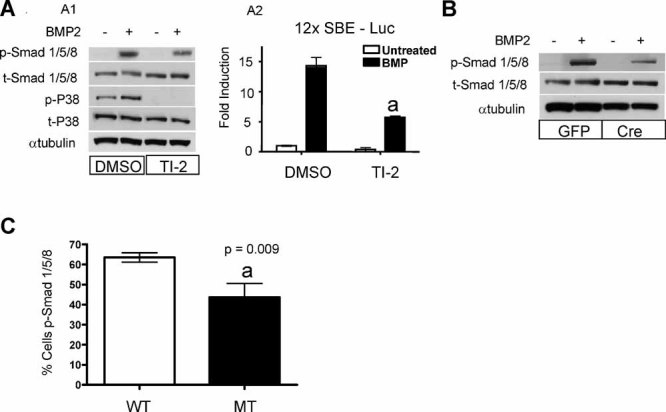
TAK1 regulates both MAPK and BMP signaling. (*A*1) Immunoblot of whole-cell lysates isolated from control and TI-2 (LLZ1640-2)–pretreated, serum-starved wild-type primary chondrocytes stimulated with BMP-2 (100 ng/mL) for 1 hour. (*A*2) 12 × -SBE luciferase assay performed on serum-starved wild-type primary sternal chondrocytes pretreated with DMSO or TI-2 and stimulated ± BMP-2 for 24 hours. (*B*) Immunoblot from primary sternal chondrocyte whole-cell lysates after Ad-Cre-mediated deletion of TAK1. (*C*) Quantification of pSmad 1/5/8 IHC on humerus of E14.5 wild-type and *Tak1^Col2^* embryos (^a^*p* < .05 versus DMSO + BMP-2 control).

To identify the potential molecular mechanisms underlying the impaired BMP responsiveness and chondrocyte maturation observed within TAK1-inhibited or -deficient cultures, we first performed studies analyzing the activation of canonical and noncanonical BMP signaling targets Smad 1/5/8 and p38, respectively. Treatment of sternal chondrocytes with TAK1 inhibitor TI-2 (LLZ1640-2, 3 µM) resulted in decreased Smad 1/5/8 and p38 phosphorylation (Fig. 4*A*, *A*1), as well as decreased luciferase activity from the BMP-responsive reporter 12 × -SBE (Fig. 4*A*, *A*2). To further support our findings, we verified that loss of *Tak1* via *Cre*-mediated deletion decreased BMP-2-induced phosphorylation of Smads 1/5/8 (Fig. 4*B*). Additionally, treatment of RCS cells with varying concentrations of TI-2 demonstrated a dose-response for TAK1 regulation of Smad 1/5/8 phosphorylation, *noggin* expression, and the induction of the 12 × -SBE luciferase reporter (Supplemental Fig. 4*C*). Collectively, these data suggest that TAK1 affects both canonical and noncanonical BMP signaling in vitro.

To directly assess the effect of *Tak1* deletion on canonical BMP signaling in vivo, we performed IHC for p-Smad 1/5/8 on histologic sections of the humerus from E14.5 wild-type and *Tak1* mutants. Analyses identified a significant decrease (∼32%) in the number of p-Smad 1/5/8 staining nuclei in the proliferating and prehypertrophic chondrocyte regions of *Tak1* mutants (Fig. 4*C*). Furthermore, in situ hybridization of *Tak1* mutant growth plates exhibit reduced levels of the BMP target gene *Id1* (data not shown). These data strongly suggest that TAK1 plays an important role in regulating the BMP signaling pathway during chondrocyte maturation in vivo.

### *Tak1*^*Col2*^ and *Tak1*^*Prx*^ mutants exhibit additional defects in joint and skeletal development

**Fig. 5:**
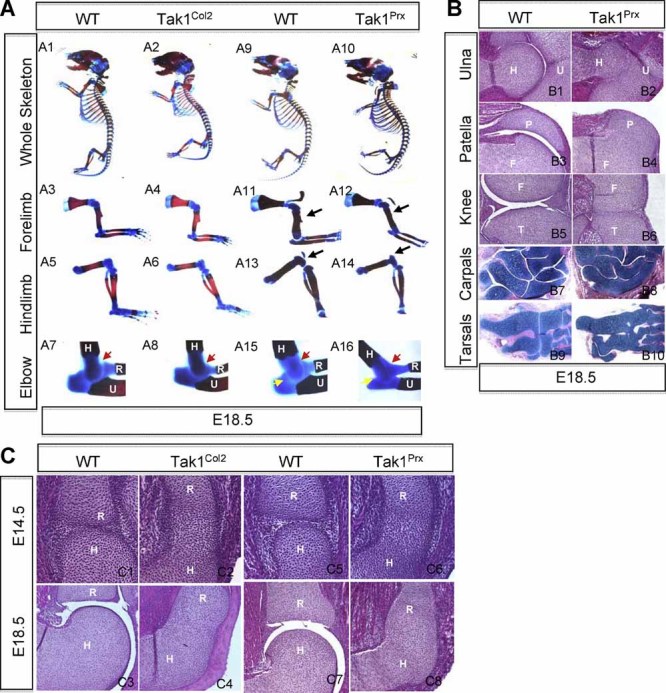
TAK1 is required for normal skeletal development and joint morphogenesis. (*A*) Alcian blue and alizarin red staining of E18.5 wild-type, *Tak1^Col2^*, and *Tak1^Prx^* embryos. Black arrow (*A*11, *A*12) indicates deltoid tuberosity and fusion of the patella (*A*13, *A*14) and humerus (H), radius (R), and ulna (U) at the elbow (*A*7, *A*8, *A*15, *A*16). Red arrows (*A*7, *A*8, *A*15, *A*16) indicate fusion of humerus and radius. (*A*15, *A*16) Yellow arrows indicate lateral fusion of the humerus (H) and ulna (U). (*B*) H&E staining of E18.5 wild-type and *Tak1^Prx^* embryos at the lateral humeroulnar joint (*B*1, *B*2), the femorpatellar joint (*B*3, *B*4), and the lateral aspect of the knee joint (*B*5, *B*6). (*B*7–*B*10) Alcian blue, hematoxylin, and orange G staining of carpals (*C*5, *C*6) and tarsals (*C*7, *C*8) at E18.5 in wild-type and *Tak1^Prx^* embryos. (*C*) H&E staining of the humeroradial joint of wild-type (*C*1, *C*3, *C*5, *C*7), *Tak1^Col2^* (*C*2, *C*4), and *Tak1^Prx^* (*C*6, *C*8) embryos at E14.5 (×20 magnification) and E18.5 (×10 magnification).

Whole-mount alcian blue and alizarin red staining of E18.5 *Tak1*^*Col2*^ and *Tak1*^*Prx*^ embryos revealed additional abnormalities during skeletal development caused by the tissue-specific deletions of *Tak1*. Targeted deletion of *Tak1* in type II collagen–expressing cells resulted in a shorter axial skeleton, a domed-shape skull, a smaller ribcage with delayed chondrocyte maturation, cervical vertebral fusions, and a single joint fusion of the radius and humerus within the appendicular skeleton (Fig. 5*A*, *A*1 through *A*8 and Supplemental Fig. 3*A, B*). Deletion of *Tak1* in the early limb mesenchyme did not alter the size of the ribcage, although it did result in an incomplete fusion of the sternum at midline (Supplemental Fig. 3*C*). The axial skeleton was not significantly affected in these mutants (Fig. 5*A*, *A*9 and *A*10) because most axial cartilages are not targeted by the *Prx1Cre* transgene. *Tak1*^*Prx*^ embryos did, however, exhibit humeroradial fusions in the forelimbs (Fig. 5*A*, *A*15 and *A*16) identical to those observed in *Tak1*^*Col2*^ embryos. Skeletal staining of *Tak1*^*Prx*^ embryos also exhibited an absence of the deltoid tuberosity (Fig. 5*A*, *A*11 and *A*12), patellar fusions of the knee (Fig. 5*A*, *A*13 and *A*14), and multiple other joint fusions in both the forelimbs and hindlimbs (Fig. 5*A*, *A*11 through *A*16, *C, C*1 through *C*8, and data not shown). Histologic examination of *Tak1*^*Prx*^ limb sections at E18.5 highlights additional fused-cartilage elements, including frequent humeroulnar fusions (Fig. 5*B*, *B*1 and *B*2), patellar knee fusions (Fig. 5*B*, *B*3 and *B*4), occasional lateral knee fusions (Fig. 5*B*, *B*5 and *B*6), fusion of the carpals (Fig. 5*B*, *B*7 and *B*8), and fusion of tarsal elements (Fig. 5*B*, *B*9 and *B*10). Of note, *Tak1*^*Prx*^ mutant knees also lacked histologically defined synovial or meniscal tissues at E18.5 (Fig. 5*B*, *B*3 and *B*4, and data not shown). Since the elbow joints for both *Tak1*^*Col2*^ and *Tak1*^*Prx*^ embryos displayed cartilaginous fusions, we compared histologic sections from the mutant and wild-type joints at E14.5 and E18.5 to determine the onset and extent of the defects (Fig. 5*C*, *C*1 through *C*8). At E14.5, mutant embryos showed the appearance of loose mesenchymal cells surrounding the joint region, but in place of a well-defined joint containing interzone cells, there existed only disorganized round mesenchymal-like cells that were morphologically distinct from the adjacent epiphyseal chondrocytes. (Fig. 5*C*, *C*1, *C*2, *C*5, and *C*6). By E18.5, when the joint is more mature, the *Tak1*^*Col2*^ and *Tak1*^*Prx*^ mutants lacked defined synovial tissues within and surrounding the fused elbow joints, whereas intervening round mesenchymal-like cells developed into regions of disorganized chondrocytes exhibiting little histologic staining for proteoglycans (Fig. 5*C*, *C*3, *C*4, *C*7, and *C*8, and data not shown).

**Fig. 6:**
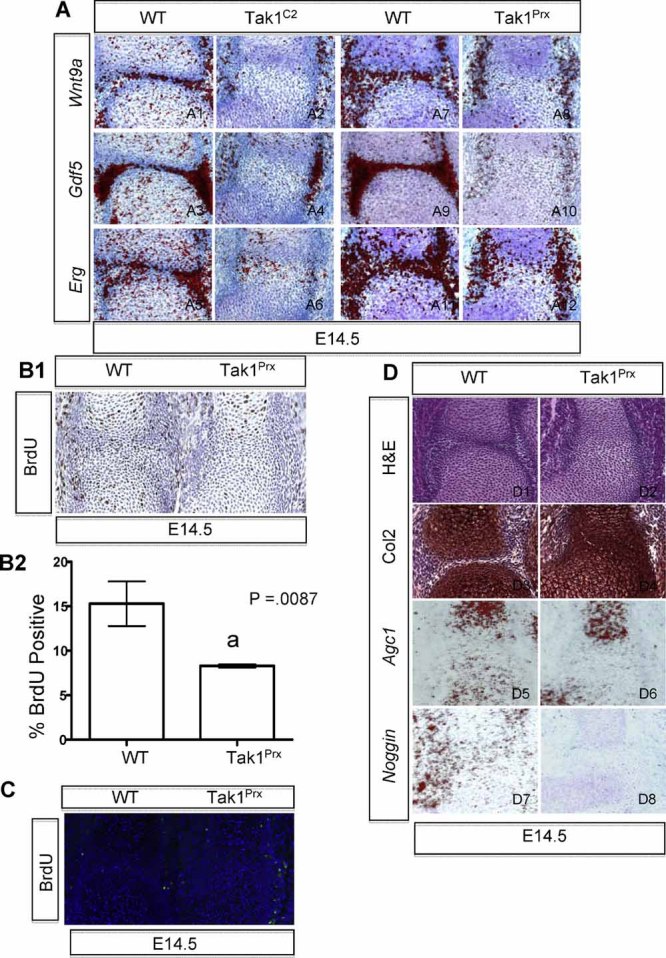
TAK1 regulates proper interzone formation/maintenance during joint development. In situ hybridization for *Wnt9a, Gdf5*, and *Erg* in the developing humeroradial joint of E14.5 *Tak1^Col2^* (*A*1–*A*6) and *Tak1^Prx^* (*A*7–*A*12) embryos. BrdU IHC of humeroradial joint of E14.5 *Tak1^Prx^* embryos (*B*1) and quantification from three sections each of wild-type and *Tak1^Prx^* embryos (*B*2). (*C*) Representative TUNEL staining of humeroradial joint in E14.5 *Tak1^Prx^* embryos. (*D*) E14.5 humeroradial joint of *Tak1^Prx^* embryos demonstrating H&E (*D*1, *D*2), *Col2* IHC (*D*3, *D*4), *Agc1* in situ hybridization (*D*5, *D*6), and *noggin* in situ hybridization (*D*7, *D*8).

To more specifically identify the cellular and molecular changes associated with the joint defects observed in *Tak1*^*Prx*^ and *Tak1*^*Col2*^ mutants, we analyzed the expression of known regulators of joint and cartilage development and quantified the proliferation and apoptosis of mesenchymal cells within the presumptive joint regions. In situ hybridization for specific regulators of joint development, *Wnt9a, Erg*, and *Gdf5* (Fig. 6*A*, *A*1 through *A*12) were performed on E14.5 sections. *Wnt9a*, a molecule with antichondrogenic activity, is normally enriched in the most medial layers of the interzone, as well as mesenchymal populations surrounding the joint space (Fig. 6*A*, *A*1 and *A*7). However, *Tak1*^*Prx*^ and *Tak1*^*Col2*^ mutant embryos displayed only weak *Wnt9a* expression in surrounding mesenchymal tissues and a complete loss of expression within the joint forming region (Fig. 6*A*, *A*2 and *A*8). *Gdf5* and *Erg*, molecules with chondrogenic activities, are normally highly expressed in multiple layers of the interzone, the articular cartilage, and within distinct wedges at the sites of cavitation (Fig. 6*A*, *A*3, *A*5, *A*9, and *A*11). Interestingly, *Tak1*^*Prx*^ and *Tak1*^*Col2*^ mutants displayed dramatically reduced *Gdf5* expression throughout the presumptive interzone, whereas *Tak1*^*Col2*^ embryos maintained some expression in the surrounding mesenchyme (Fig. 6*A*, *A*4 and *A*10). On the other hand, *Erg* expression was maintained at lower levels within cells of both the presumptive interzone and surrounding mesenchyme (Fig. 6*A*, *A*5, *A*6, *A*11, and *A*12).

To determine the cellular effects caused by misexpression of these joint regulators, we performed proliferation (BrdU) assays, apoptosis (TUNEL) assays, and in situ hybridization or immunohistochemical studies for markers of chondrocyte differentiation on joint sections from *Tak1*^*Prx*^ mice. Our data demonstrate that loss of *Tak1* in the developing joint results in reduced proliferation of the mesenchyme and chondrocytes within the presumptive joint region (Fig. 6*B*, *B*1 and *B*2), but no significant difference was observed for mesenchymal cell or chondrocyte apoptosis (Fig. 6*C*). Owing to the aberrant chondrocyte-like cell morphology found within the presumptive interzone of *Tak1*^*Prx*^ mice, we assessed the expression of chondrocyte differentiation markers on E14.5 joint sections. Immunohistochemistry for type II collagen demonstrates a continuous expression profile of the immature chondrocyte marker spanning the presumptive joint region in *Tak1*^*Prx*^ mutants, whereas wild-type mice exhibit a distinct break in type II collagen expression at the interface of opposing cartilage elements (Fig. 6*D*, *D*3 and *D*4). In situ hybridization analyses for *aggrecan1 (Agc1),* a marker of maturing chondrocytes, revealed that the immature type II collagen–expressing cells within the presumptive interzone region of *Tak1*^*Prx*^ mutants do not express *Agc1* (Fig. 6*D*, *D*5 and *D*6). Both wild-type and *Tak1*^*Prx*^ mutants demonstrate that *Agc1* expression is observed only in maturing chondrocytes adjacent to the joint region at this stage. Finally, since we identified TAK1 as a regulator of BMP signaling in chondrocytes, and since the BMP target gene *noggin* is important in joint development, we performed in situ hybridization studies to determine the effect that the absence of *Tak1* has on *noggin* expression. Compared with wild-type controls, *Tak1*^*Prx*^ mutant sections exhibit a complete absence of *noggin* expression surrounding the presumptive joint at E14.5 (Fig. 6*D*, *D*7 and *D*8). Therefore, TAK1 represents an essential regulator of interzone formation and/or maintenance, as well as, BMP signaling, during synovial joint development.

## Discussion

### TAK1 regulates chondrocyte proliferation, maturation, and apoptosis

In our model, cartilage removal of *Tak1* (*Tak1*^*Col2*^) resulted in neonatal lethality, reduced chondrocyte proliferation, reduced proliferating chondrocyte survival, and delayed chondrocyte maturation within developing embryonic cartilages. It is of note that a recent study by Shim and colleagues(34) deleted *Tak1* in chondrocytes using a different *Col2Cre* transgenic line that resulted in some similar cartilage phenotypes, except Shim and colleagues(34) observed these defects in only postnatal mutant mice. The general similarities between the two studies include decreased chondrocyte proliferation, general skeletal delay, alterations in BMP and MAPK signaling, and elbow abnormalities. Critical differences between the two models include the ability of their *Tak1*^*Col2*^ mutants to survive postnatally for 2 to 3 weeks, whereas our *Tak1*^*Col2*^ mutants die prior to birth, and the timing for the onset of cartilage phenotypes.(34) Compared with the previous study, it is likely that our mice exhibited an earlier deletion of the *Tak1* floxed alleles owing to the use of a different *Col2Cre* transgenic mouse(36,47) and therefore produced a more profound and slightly different embryonic cartilage phenotype.(34) Interestingly, our *Tak1*^*Col2*^ mutants displayed a dramatic phenotype during early stages of chondrocyte maturation (E14.5) that appeared to diminish progressively at later stages of development (E18.5). This is highlighted by the dramatic decrease in chondrocyte proliferation (∼40%) that was later restored to near-wild-type levels even in the presence of continued TAK1 expression. *Tak1*^*Col2*^ mutants also demonstrated an increase in immature chondrocyte apoptosis that was not evident in maturing chondrocytes. The combination of a transient reduction in chondrocyte proliferation and increased proliferating chondrocyte cell death may serve as an explanation for the apparent temporary delay in chondrocyte maturation. Although *Tak1*^*Col2*^ mutants maintain identifiable cartilage phenotypes until death at *P*_0_, it is difficult to ascertain whether the in vivo chondrocyte maturation phenotype is progressive or simply a reflection of the early proliferative and apoptotic defects. Our in vitro studies examining the effects of loss or inhibition of TAK1 on chondrocyte maturation suggest that the delay in chondrocyte maturation is progressive and cannot be accounted for solely by decreased proliferation and increased proliferating chondrocyte apoptosis. In these experiments, cell proliferation is reduced dramatically by days 3 to 4 in culture owing to contact inhibition, and yet the progressive delay in chondrocyte maturation, as evidenced by AP staining, is still observed in cultures well beyond this time point. Cell viability was maintained throughout the culture period and thus cannot account for the dramatically reduced levels of AP staining during the 8-day differentiation period. Collectively, these data argue that TAK1 continuously regulates the various stages of chondrocyte proliferation, maturation, and apoptosis.

In addition to deletion of *Tak1* in chondrocytes using the *Col2a1Cre* transgenic mouse, we further investigated the effects of TAK1's regulation of embryonic chondrocyte maturation via deletion of *Tak1* in the developing limb mesenchyme using the *Prx1Cre* transgenic mouse. Our additional analyses of *Tak1*^*Prx*^ mutants revealed that these embryos recapitulated the *Tak1*^*Col2*^ mutant limb cartilage phenotypes, although several additional defects were observed, including a thinner perichondrium, a disorganized and shortened columnar zone of chondrocytes, a persistent reduction in chondrocyte proliferation, and an expanded hypertrophic domain resulting from the delayed progression in chondrocyte terminal maturation. Since the *Prx1Cre* transgene targets both the chondrocytes and the surrounding perichondrium, it is likely that the additional defects observed in the growth plates of *Tak1*^*Prx*^ embryos are due to deletion of *Tak1* within perichondrial cells and an imbalance in signaling between these populations of cells. Several studies have reported the importance of the perichondrium in promoting organization of the columnar zone and regulating chondrocyte proliferation and the progression to terminal maturation. In fact, ex vivo organ culture experiments in both chick and mouse have demonstrated that local removal or experimental thinning of the perichondrium adjacent to the prehypertrophic/hypertrophic domains leads to an expansion of the hypertrophic region caused by a delay in the completion of terminal hypertrophic differentiation.(48,49) Additionally, genetic removal of several signaling pathway components, including Ihh/Pthrp,(50–52) Wnt/β-catenin,(39,53,54) and BMP/Smads(15–20) demonstrates that disruption in normal signaling events between the perichondrium and chondrocytes often results in delayed onset of chondrocyte hypertrophy and progression of terminal maturation, as well as disorganization of growth plate chondrocytes. Thus TAK1 appears to be required for normal growth plate organization and terminal chondrocyte maturation via maintenance of normal perichondrial development. The exact function of TAK1 in the perichondrium during osteoblast maturation is of great interest to our group and will be described elsewhere.

### TAK1 is required for MAPK and Smad 1/5/8 signaling during cartilage development

**Fig. 7:**
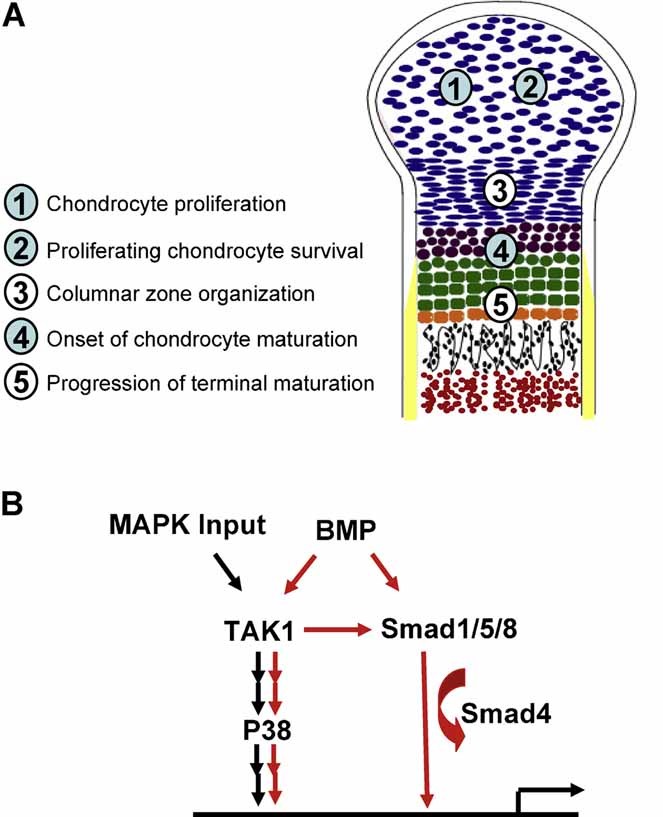
Summary of TAK1 signaling and regulation of cartilage development. (*A*) Cells are color coded as follows: Round blue cells are immature/slowly proliferating chondocytes; flat blue cells are columnar, highly proliferative chondrocytes; purple cells are prehypertrophic chondrocytes; green cells are hypertrophic chondrocytes; orange cells indicate terminally hypertrophic chondrocytes; black cells indicate trabecular osteoblasts; red cells indicate bone marrow; and the yellow area indicates maturing bone collar. Numbers in light blue circles indicate chondrocyte-specific effects of TAK1, and numbers in white circles are perichondrial effects of TAK1. (*B*) TAK1 signaling via the MAPK and BMP pathways. Red arrows indicate canonical and noncanonical BMP signaling regulation; black arrows indicate MAPK signaling via TAK1.

In addition to the role for TAK1 in activating MKK3/6 and the subsequent downstream MAPK signaling pathway component p38, recent studies have found that TAK1 is capable of interacting with all R-Smads (Smads 2/3 and Smads 1/5/8).(55) Both BMP and TGF-β pathways also can promote the activation of TAK1 via noncanonical TGF-β/BMP signaling mechanisms.(21) What remains unclear is whether the noncanonical activation of TAK1 works independently of, is required for, or signals in opposition to the TGF-β/BMP canonical signaling pathways. If TAK1 functions in a manner independent of TGF-β/BMP canonical signaling during cartilage development, one would expect that the growth plate phenotypes of our *Tak1*^*Col2*^ and *Tak1*^*Prx*^ mutants likely would resemble only those phenotypes generated by inhibition of the MAPK signaling pathway. Alternatively, if TAK1 is required for or signals in opposition to canonical TGF-β/BMP signaling, we would have expected to observe growth plate phenotypes resembling either TGF-β/BMP loss- or gain-of-function mouse models, respectively. Interestingly, we observed primarily growth plate phenotypes that were consistent with disruptions in the BMP signaling pathways during endochondral bone development. Comparison of *Tak1* mutant growth plate phenotypes with those found in *Col2Cre;Bmpr1a* ^*f/f*^*;Bmpr1b*^*+/−*^ and *Col2Cre;Smad1*^*f/f*^*;Smad5*^*f/f*^ embryos reveals striking similarities, including the delayed onset of chondrocyte hypertrophy, decreased proliferation, the rarely observed increase in proliferating chondrocyte apoptosis, disorganization of columnar zone chondrocytes, and the delayed progression of terminal maturation(16,17,20) (Fig. 7*A*). While *Col2Cre;Smad1*^*f/f*^*;Smad5*^*f/f*^ mutant phenotypes appear to be significantly more severe than our *Tak1* mutant mice, we reason that these discrepancies are likely due to only a partial loss Smad 1/5/8 phosphorylation in *Tak1*^*Col2*^ and *Tak1*^*Prx*^ mutants. It is also of note that *Col2Cre;Smad1*^*f/f*^*;Smad5*^*f/f*^ mutants show significant decreases in the expression of MAPK components TAK1 and MKK3, leading to the possibility that disruption of canonical BMP signaling also disrupts noncanonical signaling.(20) Many of the *Tak1* mutant phenotypes are recapitulated in these BMP pathway mutants, although not all the skeletal phenotypes are consistent. For example, the axial skeletal phenotypes, including fusion of cervical vertebrae in *Tak1*^*Col2*^ mutants and loss of sternal midline fusion in *Tak1*^*Prx*^ mutants, strongly resemble the phenotypes of mice deleted for *Tgfbr2* using the *Col2Cre* and *Prx1Cre* transgenes, respectively.(12,13) Additional roles for TAK1 in mediating TGF-β signals also may occur during postnatal development, although we were unable to determine whether this is the case because our mutants do not survive after birth. Studies analyzing *Col2Cre;Tgfbr2*^*f/f*^ and *Smad3*^*−/−*^ mice suggest that TGF-β plays a more prominent role in postnatal growth and maintenance of cartilage.(12,56) Finally, *Tak1*^*Col2*^ and *Tak1*^*Prx*^ mutants displayed variable MAPK loss-of-function phenotypes similar to the *dn-p38* and *ATF2*^*−/−*^ mice, although most *Tak1* mutant phenotypes were more pervasive and severe, likely owing to the fact that TAK1 is upstream of both these MAPK targets. Therefore, it seems plausible that TAK1 is an important component in the functional crosstalk among the MAPK, BMP, and TGF-β pathways in both temporal and tissue-specific contexts during skeletal development.

Our signaling studies, as well as those performed by Shim and colleagues,(34) provided further support for the importance of TAK1 in mediating BMP activation of Smad 1/5/8 and p38 via noncanonical signaling mechanisms. While the suppression of Smad 1/5/8 activation is incomplete on TAK1 inhibition, the significant decrease in activity suggests that TAK1 is at least one of the critical components by which BMP signals activate R-Smads. Interestingly, p38 activation is completely absent in both normal and BMP-induced chondrocytes following TAK1 inhibition, indicating the importance of TAK1 during normal MAPK signaling and noncanonical BMP signaling (summarized in Fig. 7*B*). The severely impaired chondrocyte maturation observed in TAK1 mutant cultures supports a role for TAK1 in the promotion of chondrocyte hypertrophy, which is in line with the function of BMP signaling on chondrocyte maturation and not that of the suppressive function for TGF-β. Additionally, TAK1 mutant chondrocytes were unable to respond to BMP-induced maturation (Fig. 3*D, E*), but the ability of TGF-β to inhibit chondrocyte maturation was well preserved (Fig. 3*D*). These data suggest that TAK1 mutant chondrocytes are less sensitive to BMP signaling and therefore are impaired in their ability to undergo chondrocyte maturation. Interestingly, studies in cardiomyocytes also have demonstrated the requirement for TAK1 in mediating BMP-induced differentiation.(57) Furthermore, our in vivo data indicate that fewer columnar and hypertrophic chondrocytes displayed significant levels of phosphorylated Smad 1/5/8 proteins in the nuclei of *Tak1*^*Col2*^ and *Tak1*^*Prx*^ mutants. It is therefore intriguing to speculate that reduced BMP/Smad signaling in the cartilage of *Tak1* mutant mice accounts for the decreased chondrocyte proliferation, increased proliferating chondrocyte apoptosis, and delayed onset of maturation.

### TAK1 regulates interzone and joint development

Defects in joint formation observed in *Tak1*^*Col2*^ and *Tak1*^*Prx*^ embryos provide direct evidence for the importance of TAK1 signaling during the process of joint formation. While several signaling molecules have been implicated in interzone formation and synovial joint development, the molecular mechanisms underlying these processes remain not well understood. Cells at the site of prospective joints form the mesenchymal interzone. Interzone cells and the surrounding mesenchymal tissues act as signaling centers expressing both antichondrogenic molecules (*Wnt9a* and *noggin*) that aid in maintaining the interzone and chondrogenic factors (*Gdf5, Erg,* and various members of the BMP family) that promote chondrocyte differentiation of the adjacent cartilage. What is clear is that an appropriate balance of these factors must be maintained in order for synovial joints to initiate and develop properly. Molecular analyses of both the *Tak1*^*Col2*^ and *Tak1*^*Prx*^ mutant joints revealed a loss of the antichondrogenic gene *Wnt9a* and *Noggin* specifically within the developing interzone. Additionally, these mutants lacked significant expression of *Gdf5*, although chondrogenic factors such as *Erg*, which is known to promote and maintain an early chondrogenic phenotype, were maintained at low levels in the surrounding joint tissue but also within the presumptive interzone. Therefore, loss of *Tak1* may lead to an imbalance of antichondrogenic and chondrogenic factors that favors chondrogenesis, the maintenance of an early chondrogenic phenotype, and the subsequent loss of interzone cells.

Analyses of the *Tak1*^*Col2*^ mutant mice revealed fusions and malformations only at the humeroradial joints, whereas *Tak1*^*Prx*^ mutants exhibited joint fusions at nearly all synovial joints except those of the digits. We believe that the elbow joint is fused within *Tak1*^*Col2*^ mutant mice owing to the promiscuity of the *Col2Cre* transgene in early mesenchymal cells of that particular joint region, as demonstrated previously using a *Col2Cre;R26R* reporter mouse.(40) *Tak1*^*Prx*^ mutant mice have more widespread defects in synovial joints because the *Prx1Cre* transgene targets nearly all mesenchymal progenitors prior to joint site determination (Supplemental Fig. 1*C*). These *Tak1*^*Prx*^ mutants strongly resemble the joint phenotypes derived from *Prx1Cre;Bmp2*^*f/f*^*;Bmp4*^*f/f*^ and *Gdf5*^*−/−*^ mutant mice, displaying joint fusions and impaired interzone formation/maintenance at nearly all synovial joints.(14) In addition, our analyses demonstrate a severe reduction in the expression of the BMP target gene *noggin* in the developing joint region; therefore, it is likely that TAK1 regulates BMP signaling not only in the growth plate but also at sites of joint formation. It is unlikely that the joint defects observed in these *Tak1* mutants are due to defective TGF-β or MAPK signaling because mutations in these signaling circuits either do not produce joint fusion abnormalities(33) or the malformations are restricted primarily to joints of the digits,(12) which are not affected in *Tak1* mutants.

In this study and through the work of Shim and colleagues,(34) we have provided the first in vivo genetic evidence that TAK1 is a critical component of both the MAPK and BMP signaling pathways during embryonic cartilage and joint development, as well as during early postnatal cartilage regulation.(34) Furthermore, our model extends the developmental analysis via use of the *Tak1*^*Prx*^ mouse model. These mice demonstrate that TAK1 signaling is not only critical for the development of nearly all synovial joints but also required in the surrounding limb mesenchyme to control several aspects of chondrocyte proliferation and differentiation. Finally, since our unique observations during skeletal development demonstrate clear similarities to the phenotypes observed in several other models of development in which BMP signaling is deficient, they confirm an in vivo physiologic role of TAK1 in BMP/Smad signaling during limb and joint development. Our continuing studies will aid in elucidating the TAK1-mediated effects on perichondrial or osteoblast maturation, axial skeletal development, and joint formation and the potential role of TAK1 in cartilage maintenance and disease.
