# Supplementary material for: TAK1 Regulates Cartilage and Joint Development via the MAPK and BMP Signaling Pathways
Source: J Bone Miner Res. 2010 Mar 8;25(8):1784–97. doi: 10.1002/jbmr.79 (PMC3153349; doi:10.1002/jbmr.79)
Supplement: Supplementary file 1 [file jbmr0025-1784-SD1.ppt]

## Slide 1
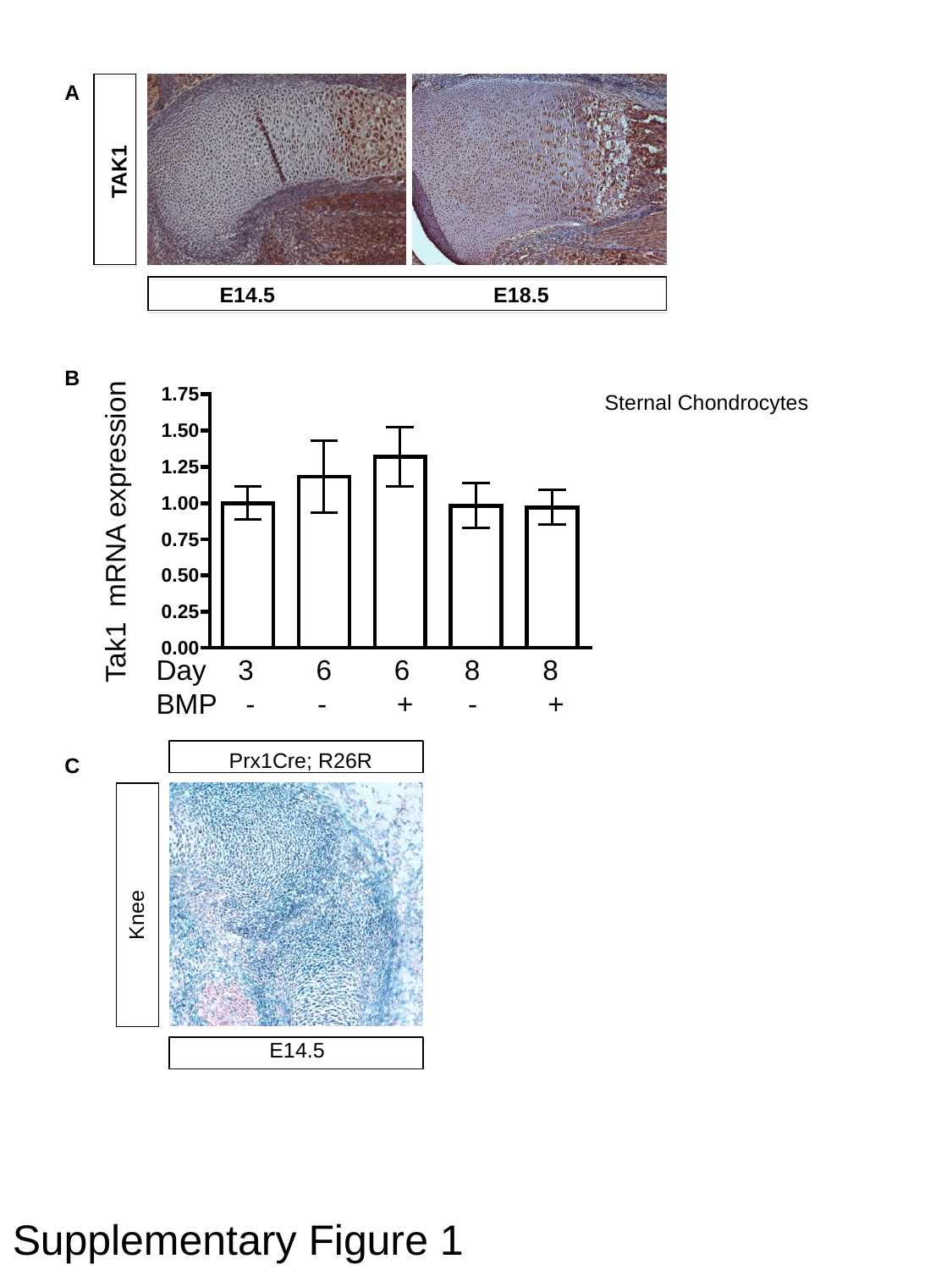

A
TAK1
 E14.5 E18.5
B
Sternal Chondrocytes
Tak1 mRNA expression
Day
BMP
 3 6 6 8 8
 - - + - +
 Prx1Cre; R26R
C
Knee
 E14.5
Supplementary Figure 1

## Slide 2
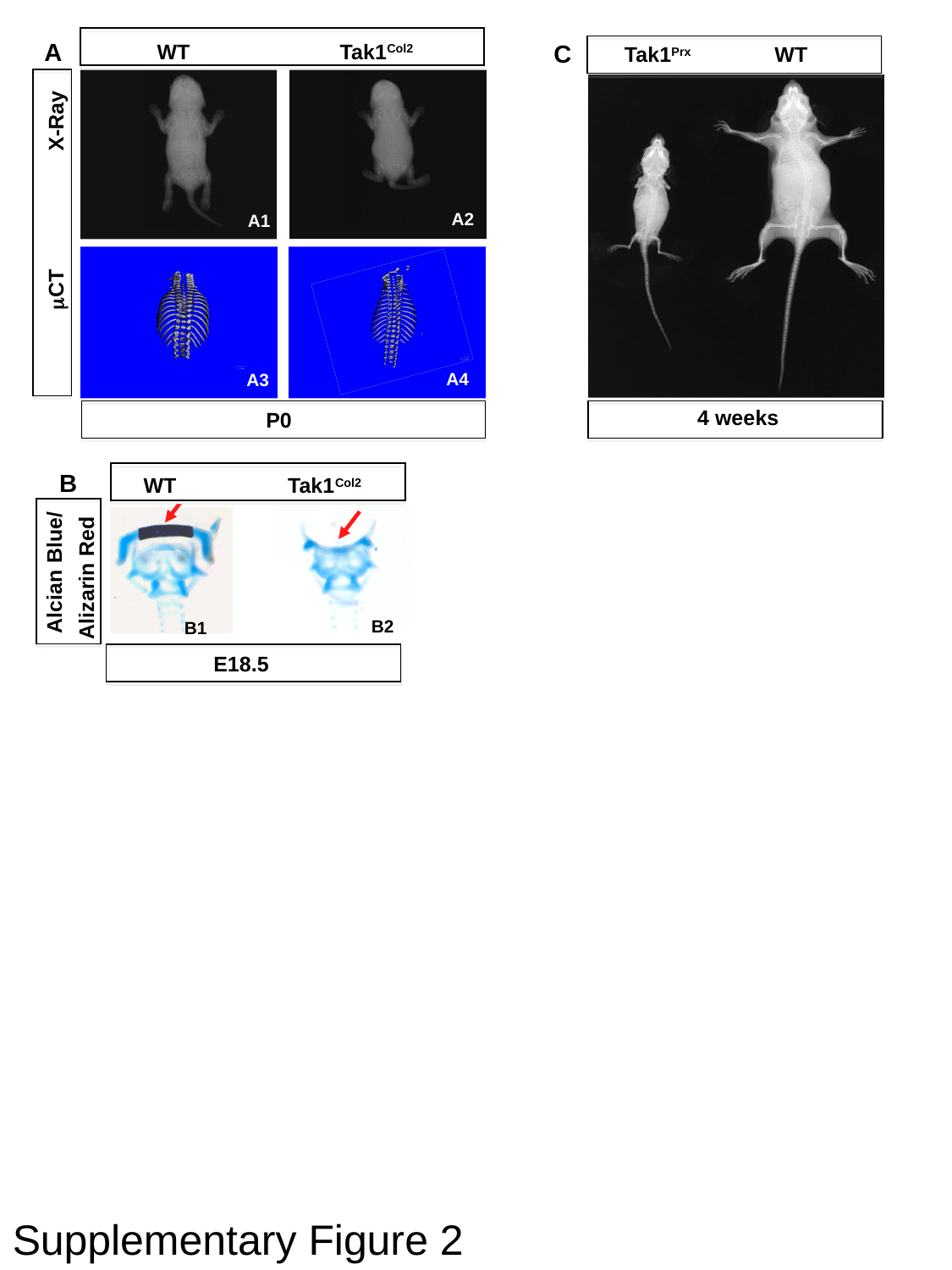

A
 WT		Tak1Col2
C
 Tak1Prx WT
  CT X-Ray
A2
A1
A4
A3
4 weeks
 P0
B
 WT	 Tak1Col2
 Alcian Blue/
Alizarin Red
B2
B1
E18.5
Supplementary Figure 2

## Slide 3
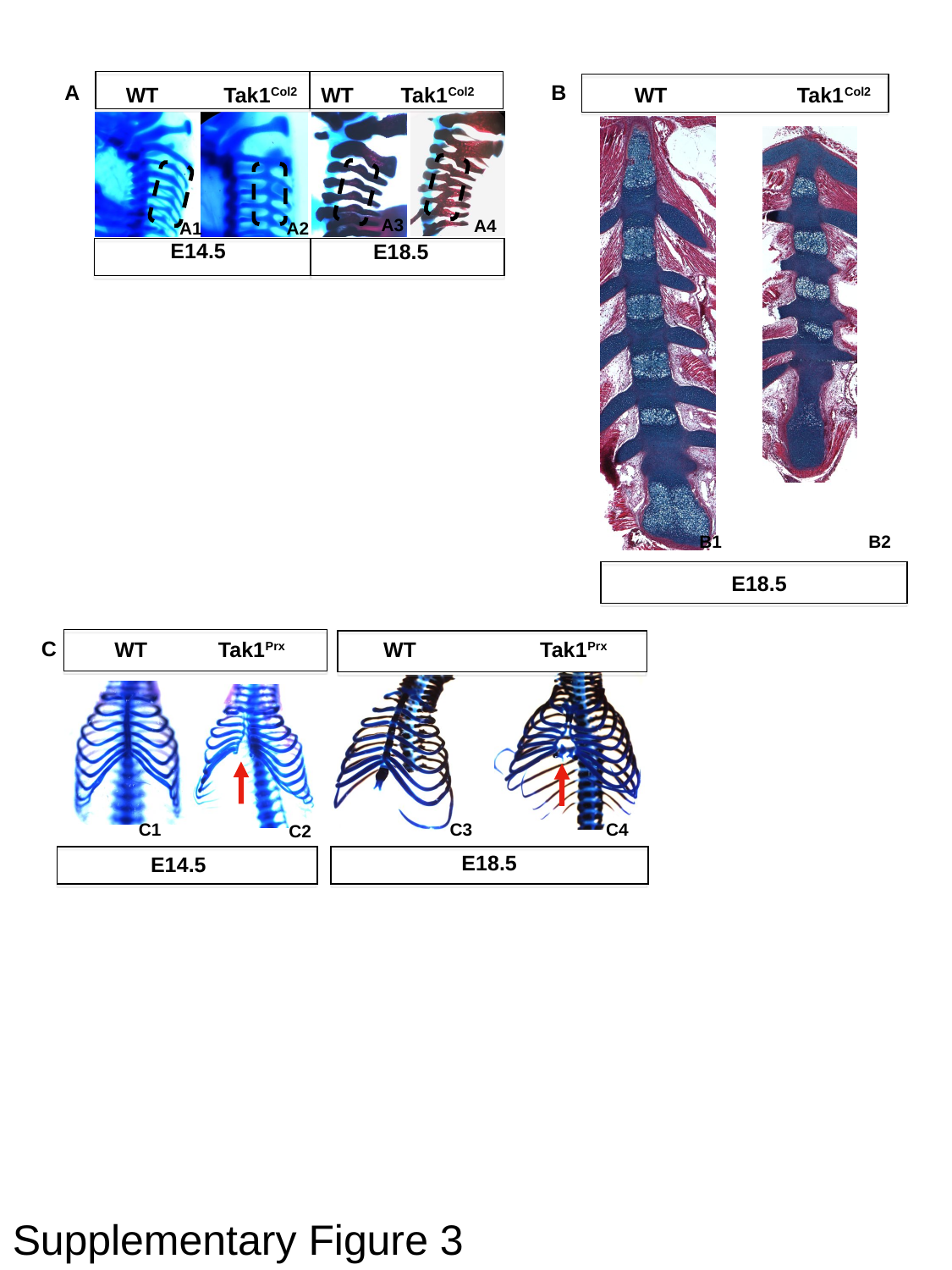

WT Tak1Col2
A
B
 WT Tak1Col2 WT Tak1Col2
A3
A4
A1
A2
E14.5
E18.5
B1
B2
 E18.5
C
 WT Tak1Prx WT Tak1Prx
C3
C1
C4
C2
E18.5
E14.5
Supplementary Figure 3

## Slide 4
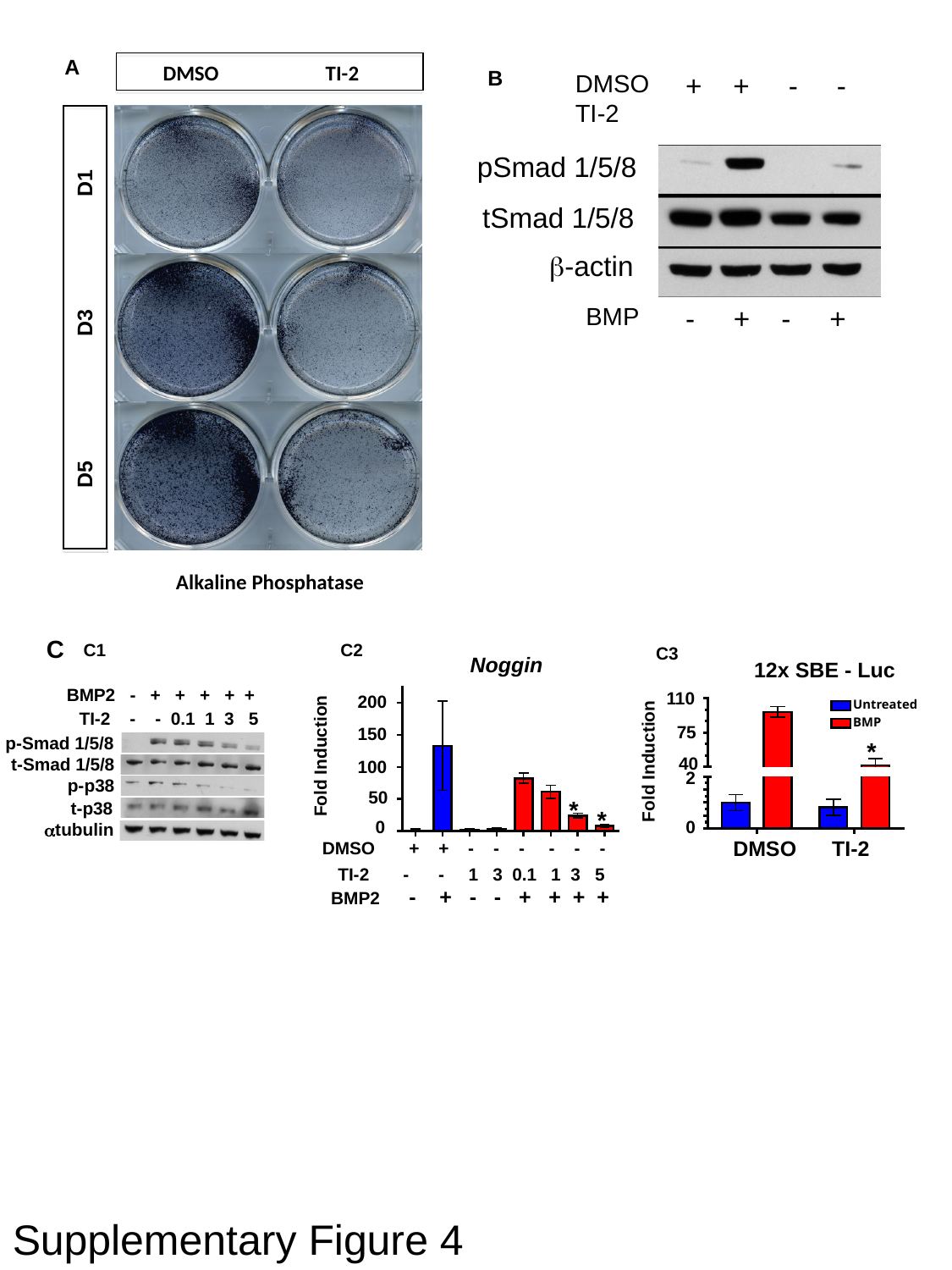

A
 DMSO TI-2
DMSO
TI-2
 + + - -
 - - + +
B
pSmad 1/5/8
tSmad 1/5/8
β-actin
BMP
 - + - +
 D5 D3 D1
 Alkaline Phosphatase
C
C2
C1
C3
Fold Induction
Fold Induction
Noggin
12x SBE - Luc
BMP2 - + + + + +
110
200
 TI-2 - - 0.1 1 3 5
75
150
p-Smad 1/5/8
*
40
t-Smad 1/5/8
100
2
p-p38
50
*
t-p38
*
0
0
αtubulin
 DMSO + + - - - - - -
 DMSO TI-2
 TI-2 - - 1 3 0.1 1 3 5
BMP2 - + - - + + + +
Supplementary Figure 4
