# Supplementary material for: TAK1 Regulates Cartilage and Joint Development via the MAPK and BMP Signaling Pathways
Source: J Bone Miner Res. 2010 Mar 8;25(8):1784–97. doi: 10.1002/jbmr.79 (PMC3153349; doi:10.1002/jbmr.79)
Supplement: Supplementary file 2 [file jbmr0025-1784-SD2.doc]

**Supplementary Figure 1:** *Tak1* temporal expression and *Prx1* joint expression. (A) TAK1 IHC on E14.5 and E18.5 humeri. (B) qPCR for *Tak1* expression in sternal chondrocyte cultures over an 8-day differentiation period +/- BMP. (C) X-gal staining of E14.5 knee joint in *Prx1Cre; R26R* embryo.

**Supplementary Figure 2:** (A) X-ray (A1, A2) and μCT (A3, A4) of P0 *Tak1^Col2^* pups. (B) Alcian blue and alizarin red staining of hyoid, larynx, and trachea of E18.5 WT and *Tak1^Col2^* embryos. (B) Red arrows indicate decreased mineralization of hyoid and fusion to the larynx. (C) X-ray of 4 week old *Tak1^Prx^* and WT mice.

**Supplementary Figure 3:** Axial skeletal defects in *Tak1^Col2^* and *Tak1^Prx^* animals. (A) Alcian blue and alizarin red staining of E14.5 (A1, A2) and E18.5 (A3, A4) cervical vertebrae. (A) Black brackets highlight region of lateral fusions in cervical vertebrae. (B) Alcian blue/H&E staining of *Tak1^Col2^* E18.5 sternum. (C) Alcian blue and alizarin red skeletal staining of E14.5 (C1, C2) and E18.5 (C3, C4) WT and *Tak1^Prx1^* mutants. (C) Red arrows indicate open sternum in *Tak1^Prx^* mutants (B2, 2B4).

**Supplementary Figure 4:** Loss of *Tak1* delays chondrocyte maturation via altered BMP signaling. (A) Alkaline phosphatase staining of chondrocytes isolated from E14.5 limbs treated for 5 days with DMSO or TAK1 inhibitor, TI-2. (b) p-Smad 1/5/8 IHC of E14.5 limb chondrocytes treated +/- BMP. (C1) Western blot of TI-2 dose response to 1 hour BMP2 treatment following serum starvation and pretreatment with DMSO or TI-2(LLZ1640-2) in RCS cells. (C2) RT-PCR for Noggin in RCS cells treated for 2 hours with BMP2 after serum starvation and pretreatment with DMSO or TI-2(LLZ1640-2). (C3) 12x-SBE Reporter assay in RCS cells pre-treated for 30 minutes with DMSO or TI-2(LLZ1640-2) and treated with or without BMP2 (100ng/ml) for 24 hours.
